# Use of mortality tables by level of deprivation in the study of social inequalities in cancer survival

**DOI:** 10.1007/s10654-024-01199-1

**Published:** 2025-02-06

**Authors:** Sarah Wilson, Ophelie Merville, Olivier Dejardin, Josephine Gardy, Quentin Rollet, Valerie Jooste, Francim Network, Florence Molinie, Laure Tron, Guy Launoy

**Affiliations:** 1 Université de Caen Normandie, ANTICIPE U1086 INSERM, 14000, University of Caen Normandy Centre Hospitalier Universitaire de Caen Bâtiment Biologie, Recherche Avenue de la Côte de Nacre CS 30001, 14033 Caen Cedex 9, France; 2 Public Health Department, Caen University Hospital, Caen, France; 3 INSERM UMR1295, Équipe EQUITY, CERPOP, Université Toulouse III, Toulouse, France; 4 FRANCIM Network, Toulouse, France; 5 Calvados Digestive Cancer Registry, University Hospital of Caen, Caen, France; 6 Calvados General Tumor Registry, Centre François Baclesse, Caen, France; 7 Registre Bourguignon Des Cancers Digestifs, Dijon University Hospital, INSERM UMR 1231, UFR Santé de Bourgogne, Dijon, France; 8 Loire-Atlantique/Vendée Cancer Registry, Nantes, France; 9 SIRIC ILIAD INCa-DGOS-INSERM-ITMO Cancer_18011, CHU Nantes, 44000 Nantes, France; 10 CERPOP, UMR 1295, Université de Toulouse III, Toulouse, France; 11 University Clinic of Hepato-Gastroenterology, Grenoble Alpes University Hospital, Grenoble, France; 12 Institute for Advanced Biosciences-INSERM U1209/ CNRS UMR 5309, University Grenoble Alpes, Grenoble, France

**Keywords:** Cancer, Net survival, Social deprivation, Mortality tables

## Abstract

**Background:**

Previous studies have reported lower net survival probabilities for socioeconomically deprived patients, using non-deprivation specific lifetables. Not accounting for the social gradient in background mortality could potentially overestimate the effect of deprivation on net survival. The aim of this study was to estimate the impact of taking into account the social gradient of expected mortality in the general population on the study of the social gradient of survival of people with cancer.

**Methods:**

French cancer registry data was analyzed, with 190,902 incident cases of nineteen cancer sites between 2013 and 2015. Deprivation was measured using the European deprivation index (EDI). Net survival was estimated thanks to additive models with French lifetables stratified on deprivation level with the EDI, using the non-parametric Pohar-perme method and flexible excess hazard modelling with multidimensional penalized splines, firstly with non-specific lifetables then with the deprivation specific-lifetables.

**Results:**

A significant effect of EDI on excess mortality hazard (EMH) remained when using the deprivation-specific lifetables for colorectal, lung cancer and melanoma in both sexes, and esophagus, bladder, head and neck and liver cancer for men, and breast, cervix and uterine cancer for women. The only site where the effect of EDI on EMH was no longer significant when using deprivation-specific lifetables was prostate cancer.

**Conclusions:**

The use of deprivation-specific lifetables confirms the existence of a social gradient in cancer survival, indicating that these inequalities do not result from inequalities in background mortality. Development of such deprivation-specific lifetables for future years is crucial to understand mechanisms of social inequalities and work towards reducing the social burden.

**Supplementary Information:**

The online version contains supplementary material available at 10.1007/s10654-024-01199-1.

## Introduction

Social disparities in cancer survival are well documented; lower survival rates have been found for patients with a lower socio-economic situation.[1–6] A social gradient in general mortality exists in many developed countries; Mackenback et al. have repeatedly found socioeconomic status measured by income, occupation and level of education to be associated with life expectancy in Europe.[7–10] Therefore distinguishing between a social gradient in mortality due to cancer and the existing social gradient in background mortality is crucial. Net survival is the survival probability that would be observed if the disease studied were the only possible cause of death. This net survival can be estimated by two main methods: the cause-specific setting using death certificate information and censoring deaths from causes other than the disease studied, and the excess hazard framework, where the study population’s death rate is compared to the mortality of the general population they belong to.[11].

Appropriate life tables are theoretically essential when studying social inequalities in mortality with the excess hazard framework. If the social gradient in the general population is not accounted for by using lifetables that aren’t socially stratified, the social gradient found in excess mortality due to a specific disease could result totally or partially from the background social gradient in mortality leading to an overestimation of the deprivation gap due to the disease studied. Socially stratified lifetables are available in multiple countries [12–16], however excess mortality should be considered in relation with the specific general population from which the study population is drawn. Previous studies tried to investigation the potential bias caused by using inappropriate lifetables using either simulated deprivation lifetables based on a subsample of the population using individual level data, or another country’s lifetables when deprivation specific lifetables are not available for the country in question.[1, 17–21] Using individual level data on a subsample of the population in the sensitivity analysis is likely to over-reduce the deprivation gap if an ecological measure is used in the study population.[22] When using other countries’ lifetables, the social stratification is not necessarily comparable between countries and the results can be difficult to translate into a public health message applicable to the country studied. Another option is to provide a correction in the modelling strategy to account for insufficiently stratified lifetables. However these different methods generally require a large population sample (over 5000) and moderate censoring rates (under 50%).[23] These limitations require extending the study period or not being able to study net survival for less common cancer sites.

Deprivation-specific lifetables have recently been developed for France, using the European Deprivation Index (EDI), an ecological measure of neighbourhood deprivation. The French version of the EDI (F-EDI) was attributed to the Permanent Demographic Sample, a large panel of the French population sampled by date of birth, and covering approximately 4% of the French population. Mortality rates by age, sex and level of deprivation for the national population were then estimated for the national population, recalibrated on national mortality rates for the same period.

Previous French studies on net survival in patients with cancer found a significant deprivation gap when using non stratified lifetables, which was then reduced for a number of cancer sites in sensitivity analyses using either English deprivation specific lifetables or simulated lifetables on a subsample of the general population.[19] The aim of this study was to estimate the impact of taking into account the social gradient of expected mortality in the general population using national deprivation-specific lifetables on the study of the social gradient of survival of people with cancer using the newly developed French deprivation specific lifetables.

## Methods

### Patient population

The French network of cancer registries (FRANCIM) provided the patient data, as described previously.[1] Patients from these registries with a diagnosis between 1 January 2013 and 31 December 2015 were included. Follow-up ended on the 30th June 2018, and patients alive at this date were censored. The information on vital status was collected through an active standardized search procedure by the French Network of Cancer Registries, based on requests to the “Répertoire National d’Identification des Personnes Physiques” [RNIPP] and, if necessary, other sources of information (including medical records or birthplace public services). The completeness and data quality of the included registries are regularly assessed by the international agency for research on cancer (IARC) and the European network of cancer registries (ENCR). This study was approved by the Commission Nationale Informatique et Libertés (CNIL – number 921057).

### Socioeconomic environment

Patients’ social environment was measured using the European deprivation index (EDI), an aggregate ecological measure of relative deprivation derived from the European Union statistics on income and living conditions survey (EU-SILC) and national census.[24] The French version of EDI is available at the IRIS level (Ilots Regroupés pour l’Information Statistique), the smallest geographical level for which census data is available, comprising approximately 2000 inhabitants. Each patient was geolocalised using Geographic Information Systems (ArgGIS) and attributed to an IRIS, and its corresponding EDI score. The EDI contains the following variables: households without access to a car, without a bath or shower, non-owners, overcrowded housing, residents with a low level of education, residents of foreign nationality, single-parent households, households with six or more persons, and unemployed residents. The index is updated throughout the years in order to best measure relative deprivation, changing the ponderations of the variables. The EDI score is a continuous variable, and the higher the score, the greater the deprivation in the IRIS.

### Statistical analyses

#### Excess death rate

An additive model was used, based on the following: the observed mortality rate in the study population (h_0_) is defined as the excess mortality hazard (EMH) due to cancer (h_E_) added to the expected mortality of the general French population (h_P_):

$$h_{0} = h_{E} + h_{P}$$

These mortality rates are defined for a given time and matched to the mortality rate of the general population by sex, age and year at death, and deprivation quintile. Excess death rate (EDR) is then considered to be mortality due to cancer, and is expressed in number of deaths per person-year.

#### Lifetables

The French lifetables used provided expected death rates by age, sex and F-EDI quintile, least deprived being q1 and most deprived q5, and in total. [25] They do not provide mortality rates by residence *Département* (which is the main territorial and administrative division in France) unlike the national lifetables produced by the National Institute of Statistics and Economic Studies (Institut National de la Statistique et des Etudes Economiques, INSEE). These life-tables covered the period 2013 to 2018, using either the 2011 or the 2015 version of the F-EDI depending on the year of census individuals in the Permanent Demographic Sample, and produced mortality rates between the ages 18 and 100 for each sex, overall and for the five quintiles of EDI. Further details on the construction of these lifetables are available in Merville et al. 2023.

The analysis was performed first using the overall expected death rate from the lifetable, then by matching this expected death rate with the death rate of the study population by F-EDI quintile.

#### Modelling strategy

The analyses were stratified by sex and by cancer site.

The logarithm of the EMH was modeled using multidimensional splines, allowing for flexible baseline hazard as well as possible non-linear and non-proportional effects of covariables. Penalization was used to reduce over-fitting of the models by using penalized one-dimensional splines and penalized tensor product splines for interactions.[26, 27].

Models were constructed step by step. If M0 was selected, the effect of EDI was considered as non-significant. If M1 was selected, the effect of EDI on the EMH was considered as significant and steady over time since diagnosis, and non-dependent of age at diagnosis. If M2 was selected, the effect of EDI was considered as significant and time-dependent but not age-dependent. If M3 was selected, the effect of EDI was considered as significant and age-dependent but not time-dependent. The non-linearity of EDI was tested in all models. The model with the lowest corrected Akaike information criteria (AIC) was selected as the best fitting model, considering a difference of at least 4 units between two AIC values. All the models were fitted using R software (4.1.2) and the survPen package (1.0.1).[28]

$$M0 :tensor\left(time since diagnosis,age at diagnosis\right)$$

- *No significant effect of deprivation on survival*
  $$M1 :M0+{s}_{1}(EDI)$$
- *There is a proportional effect of deprivation on survival (presumably non-linear)*
  $$M2 :M0+{s}_{1}\left(EDI\right)+{s}_{2}(time since diagnosis)*EDI$$
- *There is a time-dependent effect of deprivation on survival*
  $$M3 :M0+{s}_{1}\left(EDI\right)+{s}_{3}(age at diagnosis)* EDI$$
- *There is an age-dependent effect of deprivation on survival*

Net survival probabilities were computed and the deprivation gap was calculated, corresponding to the difference in net survival between the least deprived (median value of the first quintile for each sex and cancer site) and the most deprived patients (median value of the fifth quintile for each sex and cancer site), and the 95% confidence interval of this gap was obtained using the Delta method [29]. The gap was considered to be statistically significant if the associated 95% confidence interval did not contain 0. The adequacy of the selected model was also examined by comparing the net survival probabilities predicted by the model and those derived from a non-parametric method (Pohar Perme) [11] and the “relsurv” package.

A complete case analysis was performed (n = 190,902 cases), cases with missing EDI were excluded (n = 2,089 (1.1% of the overall study population)).

## Results

A total of 190,902 cases were included in the study, of which 102,954 (53.9%) concerned men and 87,948 (46.1%) women. Table 1 presents age and year of diagnosis, as well as EDI distribution by cancer site.

**Table 1:** Population characteristics by cancer site and sex

Cancer sites	N	Age at diagnosis Median (Q1–Q3)	Year of diagnosis			EDI Median (Q1–Q3)
			2013	2014	2015	
*Men*						
Prostate	28,985	69 (64, 76)	9387	9413	10,185	− 1.0 (− 2.7, 1.0)
Colon-rectum	15,033	72 (63, 80)	5011	5174	4848	− 1.0 (− 2.5, 1.1)
Oesophagus	2794	67 (60, 76)	960	979	855	− 0.8 (− 2.4, 1.3)
Stomach	3044	72 (62, 81)	1096	1037	911	− 0.9 (− 2.5, 1.2)
Lung	17,109	67 (60, 76)	5712	5826	5571	− 0.4 (− 2.2, 1.9)
Pancreas	3918	70 (62, 79)	1273	1274	1371	− 0.9 (− 2.6, 1.2)
Skin melanoma	3784	66 (53, 76)	1197	1304	1283	− 1.3 (− 3.0, 0.6)
Sarcoma	748	67 (54, 78)	226	276	246	− 1.1 (− 2.9, 0.8)
Bladder	5883	74 (66, 82)	2027	1950	1906	− 0.8 (− 2.5, 1.4)
Head and neck	7923	63 (56, 71)	2706	2665	2552	− 0.3 (− 2.2, 2.1)
Central nervous system	1789	65 (54, 74)	585	603	601	− 1.0 (− 2.7, 1.2)
Thryoid	1515	57 (46, 67)	521	498	496	− 1.0 (− 2.8, 1.2)
Kidney	4454	66 (58, 75)	1400	1515	1539	− 0.9 (− 2.6, 1.2)
Liver	5136	69 (63, 77)	1713	1773	1650	− 0.8 (− 2.5, 1.4)
Biliary ducts	839	75 (66, 82)	259	274	306	− 1.0 (− 2.7, 0.9)
*Women*						
Cervix	1883	53 (43, 67)	590	662	631	− 0.2 (− 2.3, 2.3)
Uterus	4596	69 (62, 78)	1568	1542	1486	− 0.7 (− 2.5, 1.5)
Ovary	3037	68 (60, 78)	1010	1034	993	− 0.8 (− 2.6, 1.4)
Breast	33,535	63 (52, 73)	10,989	11,476	11,070	− 0.9 (− 2.6, 1.3)
Colon-rectum	12,873	75 (64, 84)	4,299	4,313	4,261	− 0.7 (− 2.4, 1.4)
Oesophagus	737	72 (62, 82)	242	245	250	− 0.6 (− 2.5, 1.6)
Stomach	1707	77 (63, 85)	600	570	537	− 0.6 (− 2.3, 1.7)
Lung	7111	65 (57, 76)	2256	2410	2445	− 0.4 (− 2.3, 2.0)
Pancreas	3776	76 (66, 84)	1267	1287	1222	− 0.7 (− 2.5, 1.4)
Skin melanoma	3880	61 (47, 74)	1261	1316	1305	− 1.2 (− 2.9, 0.8)
Sarcoma	621	66 (55, 79)	211	190	220	− 0.8 (− 2.7, 1.4)
Bladder	1344	79 (68, 86)	443	484	417	− 0.4 (− 2.3, 1.8)
Head and neck	2326	64 (56, 76)	768	828	730	− 0.2 (− 2.2, 2.1)
Central nervous system	1444	67 (56, 78)	472	497	475	− 1.0 (− 2.4, 1.4)
Thryoid	4682	53 (41, 64)	1551	1613	1518	− 0.9 (− 2.7, 1.3)
Kidney	2180	69 (59, 79)	746	751	683	− 0.7 (− 2.5, 1.5)
Liver	1290	74 (65, 82)	429	441	420	− 0.4 (− 2.3, 2.0)
Biliary ducts	926	79 (69, 86)	297	334	295	− 0.6 (− 2.4, 1.4)

### Comparison of models selected depending on the lifetables used

For all the following sites, model selection was identical for non-specific and deprivation-specific lifetables. A proportional effect of deprivation on excess mortality hazard (EMH) was found for both sexes for colon rectum cancer and melanoma (Table 2). For men, esophagus, bladder and head and neck were sites where a proportional effect of EDI was found. For women, deprivation had a proportional effect on EMH for lung, breast, cervix and corpus uteri. An age-dependent effect was found for lung cancer in men, and a time-dependent effect was found for liver cancer also in men.

**Table 2:** Effect of EDI on net survival for men and women by cancer site, using non-specific lifetables and using deprivation-specific lifetables

	Men		Women	
	Non-specific lifetables	Deprivation-specific lifetables	Non-specific lifetables	Deprivation-specific lifetables
Breast	–	–	Proportional	Proportional
Prostate	Age-dependant	No effect	–	–
Colon-rectum	Proportional	Proportional	Proportional	Proportional
Oesophagus	Proportional	Proportional	No effect	No effect
Stomach	No effect	No effect	No effect	No effect
Lung	Age-dependant	Age-dependant	Proportional	Proportional
Pancreas	No effect	No effect	No effect	No effect
Skin melanoma	Proportional	Proportional	Proportional	Proportional
Sarcoma	No effect	No effect	No effect	No effect
Bladder	Proportional	Proportional	No effect	No effect
Head and neck	Proportional	Proportional	No effect	No effect
Central nervous system	No effect	No effect	No effect	No effect
Thryoid	No effect	No effect	No effect	No effect
Kidney	No effect	No effect	No effect	No effect
Liver	Time-dependant	Time-dependant	No effect	No effect
Biliary ducts	No effect	No effect	No effect	No effect
Cervix	–	–	Proportional	Proportional
Uterus	–	–	Proportional	Proportional
Ovary	–	–	No effect	No effect

No effect: M0 selected; proportional: M1 selected; Time-dependent: M2 selected; Age-dependent: M3 selected

For both sexes, no effect of EDI on EMH was found for stomach cancer, pancreatic cancer, sarcoma, central nervous system cancer, thyroid, kidney and biliary duct cancer. In addition for women, no effect was found for esophageal, bladder, head and neck, liver and ovarian cancer.

Prostate cancer was the only site where a significant effect of deprivation was found using non-specific lifetables (at 5 years DG = 3.2 [2.1; 4.2]) and no effect was found when using deprivation-specific lifetables (at 5 years DG = 0.39 [− 0.0007; 0.86]]).

### Variation between Q1 and Q5 and 5-year deprivation gap

*The results are presented for the most common cancer sites, results for the remaining sites are presented in supplementary data.*

**Fig. 1:**
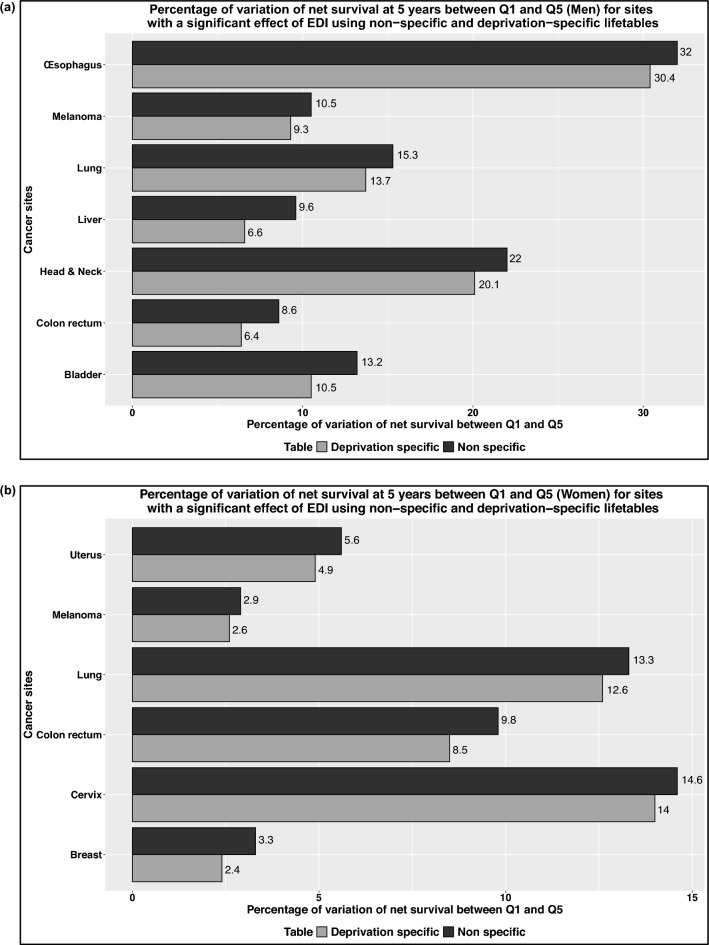
Percentage of variation of net survival at 5 years between Q1 and Q5 for sites with a significant effect of EDI using non−specific and deprivation−specific lifetables for men (Fig. 1a) and women (Fig. 1b)

- A significant and proportional effect of EDI on EMH was found for colorectal, esophageal, head and neck, CNS cancer and melanoma in men. The deprivation gap (DG) was reduced for all these cancer sites when using deprivation specific lifetables but remained significant.
  For colorectal cancer at 5 years DG = 5.8 [3.3;8.2] with non-specific tables and DG = 4.2 [1.9;6.6] with deprivation-specific lifetables (Table 3a) corresponding to a percentage of variation of net survival probability between Q1 and Q5 at 5 years of 8.6% with non-specific lifetables and 6.4% with deprivation-specific lifetables (Fig. 1a). For lung cancer for an age at diagnosis of 67 (median diagnosis age) DG = 3.9 [2.2;5.5] with non-specific lifetables and 3.5 [1.8;5.1] with deprivation-specific lifetables corresponding to a percentage of variation of net survival probability between Q1 and Q5 at 5 years of 15.3% with non-specific lifetables and 13.7% with deprivation-specific lifetables. For an age at diagnosis of 80, the deprivation gap was no longer statistically significant (Table 3a). The largest DG found was for head and neck cancer, at 5 years DG = 11.4 [8.0; 14.7] with non-specific lifetables and 10.2 [6.9; 13.5] with deprivation-specific lifetables.
- A significant and proportional effect of EDI on EMH was found for breast, colorectal, lung, cervical and uterine cancer as well as melanoma for women. The deprivation gap was reduced for all these cancer sites when using deprivation specific lifetables but remained significant.
  For colorectal cancer at 5 years DG = 6.6 [4.0;9.1] with non-specific tables and DG = 5.6 [3.1;8.2] with deprivation-specific lifetables (Table 3b) corresponding to a percentage of variation of net survival probability between Q1 and Q5 at 5 years of 9.8% with non-specific lifetables and 8.5% with deprivation-specific lifetables (Fig. 1b). For lung cancer, DG = 4.3 [1.6;7.1] with non-specific lifetables and 4.1 [1.3; 6.8] with deprivation-specific lifetables corresponding to a percentage of variation of net survival probability between Q1 and Q5 at 5 years of 13.3% with non-specific lifetables and 12.6% with deprivation-specific lifetables. For breast cancer, DG = 3.1 [2.2;4.0] with non-specific lifetables and 2.3 [1.4;3.1] with deprivation-specific lifetables corresponding to a percentage of variation of net survival probability between Q1 and Q5 at 5 years of 3.3% with non-specific lifetables and 2.4% with deprivation-specific lifetables. For women, the largest DG found was for cervix cancer, at 5 years DG = 9.6 [4.5; 14.7] with non-specific lifetables and 9.1 [4.1; 14.1] with deprivation-specific lifetables.

The non-parametric analyses supported the main analyses results (eTable 1); net survival rates were comparable when using non-specific tables and deprivation-specific tables using both non-parametric and parametric methods.

**Table 3:** Deprivation gap at 1 and 5 years in men, using deprivation-specific and non-deprivation specific lifetables according to the selected model

		Deprivation gap at 1 year		Deprivation gap at 5 years	
		Non-specific lifetable	Deprivation-specific lifetable	Non-specific lifetable	Deprivation-specific lifetable
Colon rectum		2.8 [1.6;3.9]	2,0 [0,9; 3,2]	5.8 [3.3; 8.2]	4.2 [1.9; 6.6]
Oesophagus		6.3 [2.9; 9.8]	5.9 [2.5; 9.2]	6.0 [2.7; 9.3]	5.6 [2.4; 8.8]
Melanoma		2.5 [1.3; 3.7]	2.2 [1.0; 3.4]	10.2 [6.3; 14.2]	8.9 [5.1; 12.8]
Lung	Age = 67	3.9 [2.2; 5.6]	3.5 [1.8; 5.2]	3.9 [2.2; 5.5]	3.5 [1.8; 5.1]
	Age = 80	2.4 [0.1; 4.7]	1.9 [− 0.4; 4.2]	1.9 [0.1; 3.7]	1.5 [-0.3; 3.3]
Liver		3.7 [0.6; 6.9]	3.4 [0.2; 6.5]	2.1 [− 1.6; 5.7]	1.5 [− 2.2; 5.1]
Head and neck		6.2 [4.3; 8.1]	5.6 [3.7; 7.4]	11.4 [8.0; 14.7]	10.2 [6.9; 13.5]
Bladder		4.1 [1.9; 6.3]	3.2 [1.1; 5.3]	7.4 [3.5; 11.3]	5.8 [2.1; 9.5]

	Deprivation gap at 1 year		Deprivation gap at 5 years	
	Non-specific lifetable	Deprivation-specific lifetable	Non-specific lifetable	Deprivation-specific lifetable
Colon rectum	3.3 [2.0; 4.6]	2.8 [1.5; 4.1]	6.6 [4.0; 9.1]	5.6 [3.1; 8.2]
Melanoma	0.5 [0.1; 0.8]	0.5 [0.1; 0.8]	2.8 [1.1; 4.4]	2.5 [0.7; 4.2]
Lung	3.6 [1.3; 5.9]	3.4 [1.1; 5.7]	4.3 [1.6; 7.1]	4.1 [1.3; 6.8]
Uterus	1.6 [0.6; 2.6]	1.4 [4.2; 2.4]	4.4 [1.7; 7.1]	3.9 [1.1; 6.6]
Cervix	3.3 [1.5; 5.1]	3.1 [1.4; 4.9]	9.6 [4.5; 14.7]	9.1 [4.1; 14.1]
Breast	0.6 [0.4; 0.9]	0.5 [0.3; 0.7]	3.1 [2.2; 4.0]	2.3 [1.4; 3.1]

## Discussion

Aside from for prostate cancer, the results were stable between the two analyses; a significant effect of deprivation on EMH remained when using deprivation specific lifetables for all cancer sites where a significant effect was present using non-specific lifetables. Previous studies suggested an overestimation of the deprivation gap when using non deprivation specific lifetables, and whilst this overestimation is mathematically present in this study, the variation in net survival is very small. These results suggest, excluding prostate cancer, that the social gradient observed in cancer net survival is primarily due to the social determinism in prognostic factors in cancer, and less from other causes of death potentially also socially determined.[30–32].

When socially stratified lifetables are not available at a national level, sensitivity analyses using simulated lifetables from a subsample of the population or another country’s lifetables have been employed. A study on net survival in numerous cancer sites in Japan found a smaller deprivation gap when using lifetables based on England’s deprivation pattern on mortality rates, which remained significant for both men and women for all cancer sites where a significant effect was initially found with non-specific lifetables.[17] On the contrary, other studies found an absence of effect of deprivation on various cancer sites when using simulated deprivation-specific lifetables. [1, 20] This could result from using different measures of social environment, such as Antunes et al. who found a lack of effect of EDI when using lifetables with education level. Socioeconomic deprivation can be measured using either individual level or ecological data. At an individual level, indexes should include occupation, income and education level.[33] However, these data are not commonly available in most countries for the entire population to then create socially stratified lifetables. Therefore, ecological level data can provide information for a country’s population, such as the European deprivation index (EDI), a score produced from ten weighted variables measuring relative deprivation available at the neighborhood level.[24, 34] Moreover, the larger the geographic unit, the larger the dilution effect due to the larger population of the area and the increase in social heterogeneity. Deprivation gaps estimated with neighborhood level data have been found to be smaller than with individual level data, providing a more conservative measure.[22] Tron et al. found a reduction in effect of EDI on EMH with simulated deprivation-specific lifetables, whether with another country’s lifetables or with individual level information on income from a subsample of the French population. Therefore not taking into account deprivation in background mortality overestimates the deprivation gap in cancer mortality, however using different measures of socioeconomic environment for the study population and lifetables could potentially prevent finding an existing deprivation gap by providing an overcorrection. Grafféo et al. showed that use of lifetables lacking stratification by a variable included in the excess hazard model can result in a measurement bias in the estimate of the effect of this variable and of the other covariates in the model.[35] Thus use of stratified lifetables when available should be favored in order to correctly estimate relative survival.

The only cancer site where EDI no longer had an effect on EMH with deprivation-specific lifetables was prostate cancer. A similar result was found in the study by Tron et al. using simulated lifetables.[19] A hypothesis in their study was that the correction brought by the simulated lifetables was larger than in reality, and could have cancelled an existing effect of deprivation. This current study using the same deprivation measure for the study population and the lifetable used further contributes to the previous study by suggesting that there is in fact no significant effect of deprivation on prostate cancer net survival. This hypothesis is reinforced by the fact that prostate cancer has very high net survival rates; it is therefore plausible that the social determinism in mortality of people with prostate cancer is more linked to mortality from other causes than from prostate cancer specific mortality.[36] A study in the United States that estimated relative survival with national vs. state-specific lifetables also found surprising results for prostate cancer survival, in which black males aged 85 and older had higher prostate cancer survival than white males in the same age group. The authors found in general that among older populations (aged 85 +), state-specific lifetables were less reliable than national lifetables.[37] A spatial analysis led in the North of France on the impact of socioeconomic level measured by EDI on prostate cancer incidence, aggressiveness and mortality found both higher aggressiveness and mortality in most disadvantaged areas.[38] The proportional effect of deprivation on EMH with both nonspecific and deprivation-specific lifetables found in cases diagnosed under the age of 80 likely reflects that mortality from prostate cancer represents a larger proportion of mortality in this younger population than in older patients who have a higher likelihood of dying from other causes. However, the effect of EDI on EMH, although statistically significant, was largely reduced with deprivation-specific lifetables. Information on stage at diagnosis and the effect of deprivation on prostate cancer screening in France would be informative to further explain these associations.

This study has some limitations. The lack of information on stage at diagnosis limits the interpretation of the results, and could have helped to understand how background mortality is affected by cancer mortality especially for the results regarding prostate cancer. No socioeconomic gradient was found for several cancer sites, some sites with relatively low cancer-specific mortality (thyroid for example) and others on the contrary with relatively high cancer-specific mortality. Information on stage at diagnosis could also have been beneficial to better understand this lack of effect of deprivation on survival for these cancer sites. However this lack of stage at diagnosis applies for both the analyses (with and without deprivation-specific lifetables) and does not affect the main results of this study. The lifetables used are deprivation-specific however do not contain information on localization within France by *département*. Health outcomes have been shown to be affected by rurality, thus the lack of *département* information in the lifetables could affect background mortality.[39–42] The lifetables used are less robust for deaths under the age of 30, however few deaths were observed in the study population aged 30 and under. Although these deprivation-specific lifetables can be kept up to date, the production of such tables on a routine basis remains a challenge. However they remain the only deprivation-specific lifetables in France and therefore present a progress in the study of net survival in France.

The study does however have numerous strengths. Nineteen cancer sites were studied using a robust statistical method with flexible parametric modelling to estimate net survival, confirmed by non-parametric method. The registry-based population provides high-quality data and a large population (exhaustive of the territory covered by the registries). This is the first application of French deprivation-specific lifetables on cancer net survival using the European deprivation index. Due to its potential application in other European countries, the EDI provides a comparable measure of deprivation across European countries and if used in other European lifetables, could provide a European vision of cancer survival disparities due to deprivation. Comparison across European countries could benefit future European policies, such as Europe’s Beating Cancer plan, in order to develop successful and adapted measures to improve cancer survival.[43].

In conclusion, use of deprivation-specific lifetables confirms previous published results on net survival in cancer according to socioeconomic deprivation using non-specific lifetables, aside from results on prostate cancer for which this study brings additional clues to explain previous conflicting results. The use of deprivation-specific lifetables which are adapted to the population studied is the most accurate way to properly account for socially determined comorbidities, and other techniques tend to provide an overcorrection thus could potentially prevent finding a social gradient that exists. Further development of *département* and deprivation specific tables for future years would be beneficial to continue measuring as accurately as possible the effects of deprivation on net survival in cancer in France, crucial to continue highlighting these survival differences depending on socioeconomic status to then work towards reducing these inequalities.

## Supplementary Information

Below is the link to the electronic supplementary material.

Supplementary file1 (DOCX 17 KB)

## Data Availability

The data that support the findings of this study are available on request from the corresponding author. The data are not publicly available because of privacy or ethical restrictions.
