# Supplementary material for: Use of mortality tables by level of deprivation in the study of social inequalities in cancer survival
Source: Eur J Epidemiol. 2025 Feb 6;40(2):213–23. doi: 10.1007/s10654-024-01199-1 (PMC12018625; doi:10.1007/s10654-024-01199-1)
Supplement: Supplementary file 1 — Supplementary file1 (DOCX 17 KB) [file 10654_2024_1199_MOESM1_ESM.docx]

**eTable 1: Net survival at 5 years, using non-specific and deprivation-specific lifetables, non-parametric vs. parametric analyses**

| **Men** | | **Net survival at 5 years** | | | |
| --- | --- | --- | --- | --- | --- |
|  |  | Non-parametric | | Parametric | |
|  |  | Non-specific lifetable | Deprivation-specific lifetable | Non-specific lifetable | Deprivation-specific lifetable |
| Colon rectum | QEDI 1 | 0.65 [0.60 ; 0.70] | 0.63 [0.58 ; 0.69] | 0.68 [0.66 ; 0.69] | 0.67 [0.65 ; 0.69] |
|  | QEDI 5 | 0.61 [0.55 ; 0.67] | 0.62 [0.57 ; 0.69] | 0.62 [0.60 ; 0.64] | 0.63 [0.61 ; 0.65] |
| Esophagus | QEDI 1 | 0.29 [0.22 ; 0.39] | 0.29 [0.21 ; 0.39] | 0.22 [0.19 ; 0.26] | 0.21 [0.17 ; 0.24] |
|  | QEDI 5 | 0.19 [0.14 ; 0.27] | 0.20 [0.14 ; 0.27] | 0.16 [0.13 ; 0.19] | 0.15 [0.12 ; 0.18] |
| Melanoma | QEDI 1 | 0.98 [0.93 ; 1.04] | 0.96 [0.91 ; 1.0] | 0.97 [0.95 ; 0.98] | 0.96 [0.94 ; 0.98] |
|  | QEDI 5 | 0.92 [0.85 ; 1.00] | 0.94 [0.86 ; 1.02] | 0.87 [0.83 ; 0.90] | 0.87 [0.83 ; 0.91] |
| Lung | QEDI 1 | 0.25 [0.22 ; 0.29] | 0.24 [0.21 ; 0.28] | 0.25 [0.23 ; 0.26] | 0.24 [0.23 ; 0.26] |
|  | QEDI 5 | 0.21 [0.18 ; 0.24] | 0.21 [0.18 ; 0.24] | 0.21 [0.19 ; 0.22] | 0.21 [0.19 ; 0.23] |
| Liver | QEDI 1 | 0.23 [0.18 ; 0.29] | 0.23 [0.18 ; 0.29] | 0.22 [0.19 ; 0.25] | 0.21 [0.18 ; 0.24] |
|  | QEDI 5 | 0.20 [0.15 ; 0.26] | 0.20 [0.16 ; 0.26] | 0.20 [0.17 ; 0.23] | 0.20 [0.17 ; 0.23] |
| Head and neck | QEDI 1 | 0.53 [0.47 ; 0.60] | 0.52 [0.47 ; 0.59] | 0.52 [0.49 ; 0.54] | 0.52 [0.49 ; 0.54] |
|  | QEDI 5 | 0.36 [0.31 ; 0.41] | 0.36 [0.32 ; 0.42] | 0.40 [0.37 ; 0.43] | 0.42 [0.39 ; 0.44] |
| Bladder | QEDI 1 | 0.48 [0.40 ; 0.57] | 0.47 [0.39 ; 0.56] | 0.56 [0.53 ; 0.59] | 0.55 [0.52 ; 0.59] |
|  | QEDI 5 | 0.50 [0.43 ; 0.58] | 0.51 [0.44 ; 0.59] | 0.49 [0.45 ; 0.52] | 0.50 [0.46 ; 0.53] |

| **Women** | | **Net survival at 5 years** | | | |
| --- | --- | --- | --- | --- | --- |
|  |  | Non-parametric | | Parametric | |
|  |  | Non-specific lifetable | Deprivation-specific lifetable | Non-specific lifetable | Deprivation-specific lifetable |
| Colon rectum | QEDI 1 | 0.69 [0.63 ; 0.75] | 0.68 [0.62 ; 0.74] | 0.67 [0.65 ; 0.69] | 0.66 [0.64 ; 0.68] |
|  | QEDI 5 | 0.60 [0.55 ; 0.66] | 0.61 [0.56 ; 0.67] | 0.60 [0.58 ; 0.63] | 0.61 [0.59 ; 0.63] |
| Melanoma | QEDI 1 | 0.98 [0.93 ; 1.04] | 0.98 [0.94 ; 1.03] | 0.96 [0.95 ; 0.97] | 0.96 [0.95 ; 0.97] |
|  | QEDI 5 | 0.92 [0.84 ; 1.00] | 0.92 [0.85 ; 1.01] | 0.94 [0.91 ; 0.95] | 0.94 [0.91 ; 0.95] |
| Lung | QEDI 1 | 0.33 [0.28 ; 0.38] | 0.32 [0.27 ; 0.38] | 0.33 [0.31 ; 0.36] | 0.33 [0.30 ; 0.36] |
|  | QEDI 5 | 0.27 [0.22 ; 0.33] | 0.27 [0.22 ; 0.33] | 0.29 [0.26 ; 0.32] | 0.29 [0.26 ; 0.32] |
| Uterus | QEDI 1 | 0.81 [0.75 ; 0.87] | 0.80 [0.74 ; 0.86] | 0.79 [0.77 ; 0.81] | 0.79 [0.76 ; 0.81] |
|  | QEDI 5 | 0.71 [0.65 ; 0.78] | 0.72 [0.65 ; 0.79] | 0.74 [0.71 ; 0.76] | 0.74 [0.71 ; 0.77] |
| Cervix | QEDI 1 | 0.65 [0.54 ; 0.79] | 0.65 [0.54 ; 0.78] | 0.73 [0.69 ; 0.77] | 0.73 [0.69 ; 0.77] |
|  | QEDI 5 | 0.58 [0.49 ; 0.68] | 0.58 [0.49 ; 0.68] | 0.63 [0.58 ; 0.67] | 0.63 [0.58 ; 0.67] |
| Breast | QEDI 1 | 0.95 [0.93 ; 0.97] | 0.94 [0.92 ; 0.96] | 0.95 [0.94 ; 0.95] | 0.94 [0.94 ; 0.95] |
|  | QEDI 5 | 0.91 [0.89 ; 0.93] | 0.92 [0.90 ; 0.94] | 0.92 [0.91 ; 0.92] | 0.92 [0.91 ; 0.93] |

*The age category selected for non-parametric analyses was the decade corresponding to the median age at diagnosis (for which net survival probability is calculated using parametric modelling), in order to have a sufficient amount of cases for non-parametric analyses.*
